# Variability and individuality in the contact calls of jackdaws (*Corvus monedula*)

**DOI:** 10.1007/s10071-025-02022-4

**Published:** 2025-12-16

**Authors:** Georgine Szipl, Anton Baotic, Kurt Kotrschal

**Affiliations:** 1 Institute for Globally Distributed Open Research and Education (IGDORE), Gruenau im Almtal, Austria; 2 Acoustics Research Institute, Austrian Academy of Sciences, Vienna, Austria; 3 Department of Behavioural & Cognitive Biology, University of Vienna, Vienna, Austria

**Keywords:** *Corvus monedula*, Contact calls, Individuality, Vocal complexity

## Abstract

In socially complex species, vocal signals often convey individual identity, enabling recognition and coordination of individualized groups. Jackdaws (*Corvus monedula*) are highly social corvids that form life-long monogamous pair bonds. They frequently produce ‘tchak’ contact calls while foraging in loose ground-based heterospecific flocks where maintaining cohesion without visual contact may be advantageous. We recorded tchak contact calls from nine adult males during natural foraging and analysed their acoustic structure using both conventional acoustic methods and machine learning approaches. Unsupervised classification revealed two structurally distinct call variants, a harmonic and a noisy form, which differed in spectral and temporal properties. While the harmonic variant occurred in eight out of the nine males, the noisy form was only recorded in five individuals. Both variants encoded individual identity, and spectrogram-based analyses yielded higher classification accuracy. These results suggest that tchak calls convey stable identity cues which facilitate individual recognition. The coexistence of two structurally and individually distinctive call forms within a single call type highlights the flexibility of jackdaw vocal communication.

**Supplementary Information:**

The online version contains supplementary material available at 10.1007/s10071-025-02022-4.

## Introduction

Understanding how animals navigate complex social environments has been a central focus in animal cognition research. In group-living species, the ability to distinguish individual conspecifics provides key social advantages, such as assessing hierarchies, maintaining long-term bonds, and coordinating collective behaviours. In fact, the social domain has been proposed to drive the evolution of cognitive abilities, particularly in animals with complex social systems (Jolly 1966; Humphrey 1976). A mainstream framework to explain the evolution of complex cognition is the social brain or social intelligence hypothesis (Byrne and Whiten 1988; Dunbar 1998; de Waal and Tyack 2003), which posits that the demands of living in complex and dynamic social groups select for enhanced cognitive abilities. It was originally developed to explain the evolution of primate intelligence by linking it to their complex multilevel social structure that is constantly changing in size and composition and requires a flexible mind. This framework was successfully applied to Psittacidae (Lambert et al. 2019; Rössler and Auersperg 2023), and Corvidae birds (Emery and Clayton 2004), both known for their remarkable cognitive abilities that may have evolved – convergently to primates – to coordinate and synchronize the actions of lifelong monogamous pairs, termed ‘relationship intelligence’ (Emery et al. 2007).

Recognition of other individuals is fundamental to social communication and can occur at varying levels: class-level recognition (e.g., distinguishing mates, kin, or social group members from non-members), or true individual recognition, in which one individual is discriminated from all others (Tibbetts and Dale 2007). True individual recognition requires both signal distinctiveness and cognitive-perceptual mechanisms capable of encoding and remembering individual-specific traits (Beecher 1989). A comprehensive review by Carlson et al. (2020) summarizes empirical evidence for individual vocal recognition (IVR) in a broad range of birds and mammals, including dolphins, corvids, parrots, and primates. This review distinguishes between IVR-singular (recognition of one specific individual, e.g., a mate or neighbour) and the cognitively more demanding IVR-multiple (recognition of multiple individuals within a social group), underscoring both the functional importance and cognitive demands of individual-level discrimination. Examples include bottlenose dolphins using signature whistles (Janik et al. 2006), monk parakeets recognizing group members vocally (Berg et al. 2011), and paper wasps using visual cues for face recognition (Tibbetts 2002), all highlighting that individual recognition is not restricted to a single sensory modality. Establishing the presence of individual vocal characteristics is a crucial first step toward determining whether a species is capable of true individual recognition.

Jackdaws (*Corvus monedula*) are among the smallest corvids, but still show impressive cognitive abilities. For example, jackdaws have successfully passed tests on numerical competence (Ujfalussy et al. 2014), object permanence (Ujfalussy et al. 2013), and transitive inference (Mikolasch et al. 2013). These colonial and highly social corvids represent a compelling model for studying individual variation in vocalisations. Jackdaws form long-term monogamous pair bonds (Goodwin 1976; Henderson et al. 2000), and are organized in two social categories: resident pairs which defend a nest and breed at the same site year after year, and non-resident birds consisting of yearlings, unpaired, widowed, and non-breeding birds, as well as pairs not abiding to the same nest site (Röell 1978). Both classes mix and form flocks during foraging, breeding, and roosting (Röell 1978), providing a rich social context for vocal interactions, and potentially increasing the importance of individual recognition and memory of social relationships.

Jackdaw vocal communication plays a central role in social coordination, particularly in behaviours such as collective movement and anti-predator responses. For example, alarm (or scolding) calls, which can be distinguished by the caller’s identity, sex, and colony membership, elicit stronger recruitment responses when produced by familiar or socially close individuals (Woods et al. 2018). This suggests that jackdaws are capable of discriminating between individual callers (Coomes et al. 2019). Similarly, other types of vocalisations, including food provisioning calls (Zandberg et al. 2014) and contact calls (Stowell et al. 2016), have also been shown to be individually distinctive. Beyond individual recognition, vocal communication also facilitates group-level coordination. Playback experiments and acoustic monitoring have demonstrated that jackdaws use vocal signals to coordinate the timing of mass departures from communal roosts. In these contexts, calling intensity increases prior to synchronized take-off, indicating a form of vocal consensus-building among group members (Dibnah et al. 2022; Broad et al. 2024).

Jackdaws forage in flocks, often with sympatric species like carrion crows (*Corvus corone*) and rooks (*C. frugilegus*), and prefer agricultural landscapes due to their omnivorous feeding strategies (Röell 1978; Andren 1992). Most birds produce contact calls during foraging to maintain group cohesion while visual contact is limited (Marler 2004; reviewed in Kondo and Watanabe 2009). In jackdaws, one of the most frequently heard vocalisation is the short and high-pitched tchak contact call (Goodwin 1976), which was described to be highly variable (Lorenz 1931; Strauß 1938), and to contain individually distinct features (Stowell et al. 2016). While foraging in loose aggregations on the ground, these calls are produced regularly, suggesting that this call may play a role in short-distance coordination or recognition among foraging group members or pair partners. Whether these highly variable contact calls consist of graded variations or distinct subtypes remains unclear.

In this study, we focus on the structure of acoustic variants within tchak contact calls of jackdaws. Jackdaws are highly social birds that forage in large, fluid groups, where individuals must navigate complex social dynamics. In such contexts, contact calls may need to encode multiple layers of information – such as identity, motivational state, or group affiliation – to maintain cohesion and coordinate interactions. While previous work has shown that tchak calls can support individual recognition (Stowell et al. 2016), it remains unclear whether this individual information is preserved across acoustically variable call variants, or whether distinct call subtypes exist. Clarifying how such vocal variation is structured, and whether it reflects graded changes or discrete subtypes, can shed light on how flexible vocal systems support recognition and coordination in dynamic social groups. To address this, we recorded vocalisations from free-ranging adults during ground foraging and analysed acoustic variation across and within tchak call variants. We assess whether individual distinctiveness is maintained within subtypes and discuss the potential functional relevance of this variation for individual recognition and flock cohesion.

## Materials and methods

### Data collection and processing

Data were collected from a habituated population of free-ranging, individually marked jackdaws at the Konrad Lorenz Forschungsstelle fuer Ethologie in Austria (hereafter referred to as KLF) in Gruenau im Almtal, Upper Austria. Birds originated from five nests in 2005 and six nests in 2006, and were hand-raised at the KLF. Birds were released into free flight in June 2007; they remained in the area due to daily provisioning at a feeding station near the research station. Flock size varied during data collection, ranging from 17 to 22 identifiable individuals marked with coloured leg bands. These fluctuations were due to natural factors such as predation and migration. We used a Sony TCD-D100 Digital Audio Recorder and a Sennheiser K6 directional microphone to record tchak contact calls in October and November 2007, and February 2008. Recordings were made ad libitum; vocalisations were collected opportunistically when at least two-thirds of the flock was present, during foraging in the meadows in front of the KLF. Calling individuals were then approached and recorded. Caller identity was verbally noted on the recording immediately after each call. Due to their habituation to human presence, individuals could be approached closely, allowing for high-quality field recordings at distances of one to five meters. A total of 464 tchak calls from nine adult males were used for subsequent analyses (mean ± SD = 51.56 ± 24.87 calls per individual). Audio files were digitized at a sampling rate of 48,000 Hz with 16-bit resolution. Noise profile filtering was applied to minimize ambient noise in the recordings using Audacity v2.1.3 (Audacity Team 2014): a noise profile was created by selecting several seconds of noise at the onset of the recording of each recording session. Then, the noise reduction was applied using a 12 dB noise reduction, a sensitivity setting of 6.0, and three frequency smoothing bands. Calls were then annotated in Praat v6.4.27 (Boersma and Weenink 2016), thereby ensuring that calls were aligned at the start, assigned to the calling individual, and extracted for in-depth acoustic analysis.

### Extraction of acoustic features

Acoustic parameters were measured semi-automatically using custom-built scripts in Praat. In total, we extracted 15 parameters (Table S1 in the Supplementary Information (SI), including three temporal features (call duration, time of minimum fundamental frequency (f0), and time of maximum f0), eleven source-related features (minimum, maximum, mean, start, and end f0, f0 range, sum of variation in f0, f0 variability index, mean absolute slope (MAS), the proportion of voiced parts, jitter), and one spectral feature, the harmonic-to-noise ratio (HNR). Our parameter set, comprising temporal, source-related, and spectral features, was selected to provide a comprehensive characterization of harmonic vocalizations, guided by prior literature (Boeckle et al. 2012, 2018; Szipl et al. 2017) and by the functional relevance of these features to vocal production and perception. Due to variability in call structure, automated f0 tracking was not always reliable and was manually adjusted where necessary. The recorded tchak contact calls are short and high-pitched calls and come in numerous variations, making manual classification challenging. Machine-learning classification methods were applied to analyse both visual (spectrogram-based) and acoustic features. The same data set was used for all analyses.

### Statistical analyses

We applied a combination of unsupervised and supervised machine learning approaches to classify jackdaw contact calls and assess individual distinctiveness.

#### Unsupervised classification of Tchak calls

To identify structural variation within the tchak calls, we first applied an unsupervised classification based on spectrograms. Following Thomas et al. (2022), audio recordings of jackdaw vocalizations were bandpass-filtered between 200 and 12,000 Hz using a Butterworth filter. Then, spectrograms were created and transformed to logarithmically spaced Mel bins (hanning window, N_MELS = 40, FFT_WIN = 0.01, FFT_HOP = 1.25, overlap = 87.5%), and converted to a decibel scale with the maximum power set as a reference using the librosa package (v0.9.2) Spectrograms were stretched to equal length using phase vocoder algorithm, and z-transformed and zero-padded to obtain numeric vectors (Thomas et al. 2022). We used Uniform Manifold Approximation and Projection (UMAP; McInnes et al. 2018) for dimension reduction and visualisation (metric type = Euclidean, min_dist = 0, random_state = 42, knn = 5; umap-learn library v0.5.7). Following Xie et al. (2024), we then applied *K*-Means clustering (Scikit-learn library v0.24.2), which used Euclidean distance by default, and employed the elbow method to determine the optimal number of clusters (Syakur et al. 2018). This analysis revealed two main forms of the tchak call: a harmonic variant and a noisy variant, which served as the basis for all subsequent analyses. We used the full set of 646 tchak calls in this analysis.

#### Feature-based classification of Tchak calls

To test whether calls could be reliably classified into the two variants, and individually, by the measured acoustic features alone, we conducted feature importance analysis in the SHAP library (v0.36.0, Lundberg et al. 2020). Acoustic parameters were selected based on a SHAP value > 1; Fig. S1 in SI file). These acoustic parameters were used to train a model on 70% of the calls using eXtreme Gradient Boosting classifier (XGBoost library v1.6.0). Hyperparameters were optimized with the Optuna library (v4.2.1) using a minimization objective function over 200 trials. Five-fold cross-validation was performed within the training set during hyperparameter optimization. The final model, trained on the entire training set with the best hyperparameters, was then evaluated on the remaining 30% of calls held out as a test set. Cross-validated classification results represent the mean of the accuracy scores from each of the five folds used during cross-validation. The total set of tchak contact calls (*n* = 646) was used for this analysis.

#### Individual classification within each call variant

We then analysed the harmonic and noisy tchak calls separately to assess whether calls within each group carried individual-specific acoustic structures using supervised analyses on acoustic features and spectrograms. Using the spectrograms generated in the first part of the analysis, we applied a deep learning model using a convolutional neural network (CNN) provided by Xie et al. (2024). The CNN was executed in the TensorFlow library v2.8.0. For the acoustic features, we applied the same procedure as described above, and selected the most important features based on a SHAP value > 1 for the noisy variant and value > 2 within the harmonic variant (Fig. S2 and S3 in SI file). SHAP value thresholds for feature inclusion were set with consideration of the number of meaningful acoustic parameters available for each call variant. For the harmonic tchak, a threshold of one would have selected all but one feature (f0 range). To obtain a more limited and interpretable set of features, the threshold was increased to two. We trained models using XGBoost and optuna library for hyperparameter optimization. Learning curves showing the validation and training scores within noisy and harmonic tchak contact calls are provided in Fig. S4 and S5 in the SI file. We analysed 397 harmonic tchak calls of eight individuals, and 57 noisy tchak calls of three individuals (two individuals were removed from this analysis due to limited sample sizes for testing).

Several model evaluation metrics were used to examine model performance. We extracted precision (true positive rate), recall (model sensitivity), the f1-score (the harmonic mean of precision and recall), and support (the number of calls used for evaluation). For visualisation of the results, we drew 2D and 3D scatterplots using the UMAP coordinates with matplotlib library v3.4.3, calculated same-class probabilities, and plotted confusion matrices (seaborn library v0.11.2). All statistical analyses were performed with Python v3.9.21 (Van Rossum and Drake 2009). Figures of example spectrograms were plotted using RStudio (v2024.12.1 + 563) with R v4.5.0 (R Core Team 2018), using tuneR (Ligges et al. 2023), the seewave library (Sueur et al. 2008), ggplot2 (Wickham 2016), the viridis package (v0.6.5, Garnier et al. 2024), and the cowplot package (v1.1.3, Wilke 2024).

## Results

### Contact call classification based on spectrograms and acoustic features

**Fig. 1:**
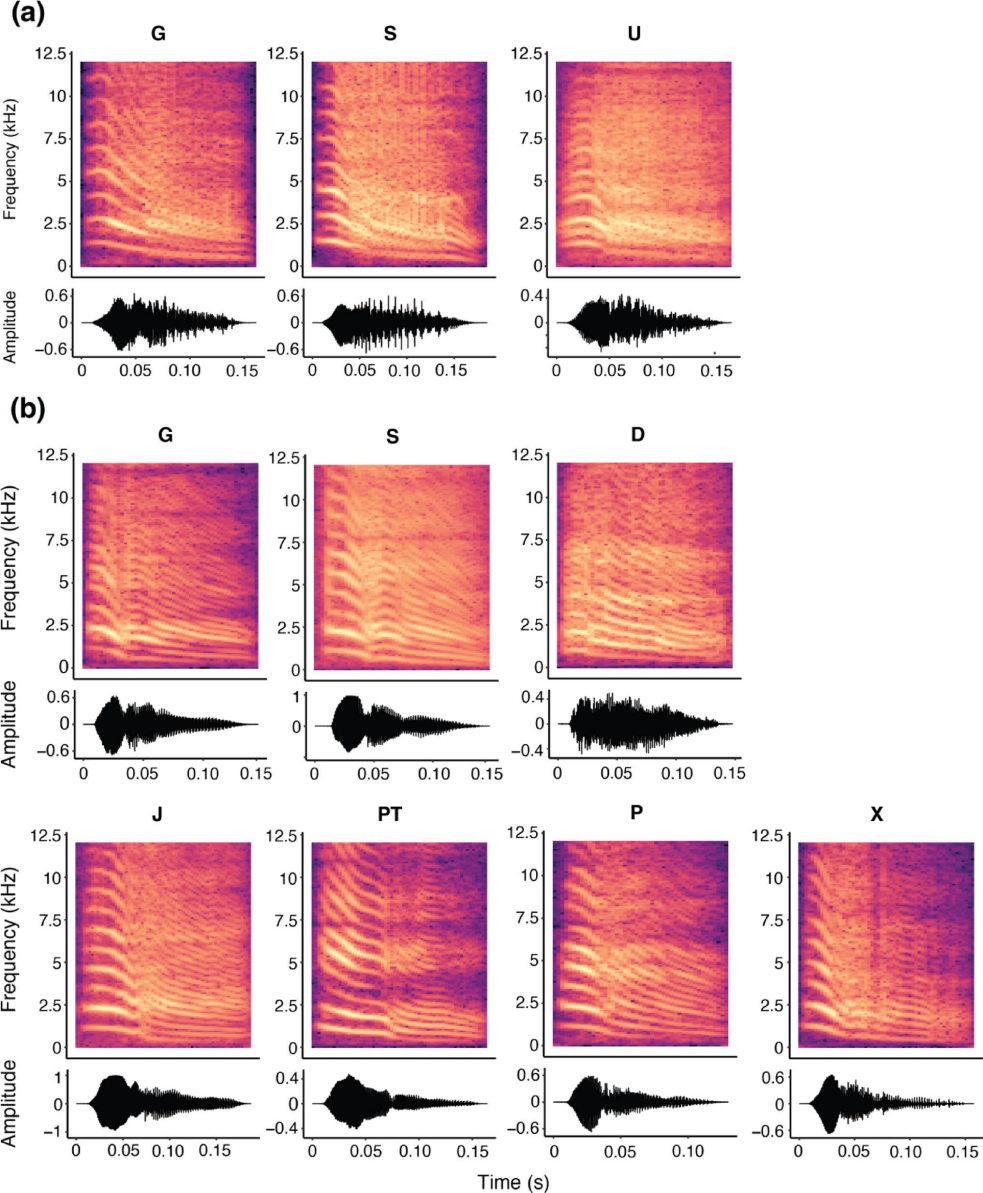
Representative spectrograms and waveforms of (**a**) noisy and (**b**) harmonic tchak contact calls produced by nine individual jackdaws. Capital letters indicate individual identity. Noisy tchak calls are characterized by low harmonicity and numerous time-frequency modulations towards the end of the call. Harmonic calls show a regular, periodic structure with a clearly visible fundamental frequency and multiple harmonic bands

The tchak contact call is a short, high-pitched, and harmonic call with an average duration of 157 ± 22ms (SD) and a mean fundamental frequency at 709.24 ± 116.15 Hz (Supplementary Table S2 in SI file). The clustering of the contact calls based on the spectrograms suggested three clusters. The first cluster contained calls that were characterized by low harmonicity, more broadband spectral energy, and a highly time-frequency modulated end (Fig. 1a) and were therefore categorized as ‘noisy tchak’ calls. Noisy calls started with a segment dominated by a harmonic structure and a visible fundamental frequency which became increasingly noisy toward the end of the call. The remaining calls of cluster two and three were more harmonic and tonal variants of the tchak call. These two clusters were considered one variant, and the calls were termed ‘harmonic tchak’ calls. Harmonic calls show a regular, periodic structure with clearly visible f0 and multiple harmonic bands (Fig. 1b). The f0 contour typically exhibits a gradual downward modulation, often with a brief inflection marking a transition between two segments of the call. Waveforms reflect consistent voicing and gradual amplitude modulation.

**Fig. 2:**
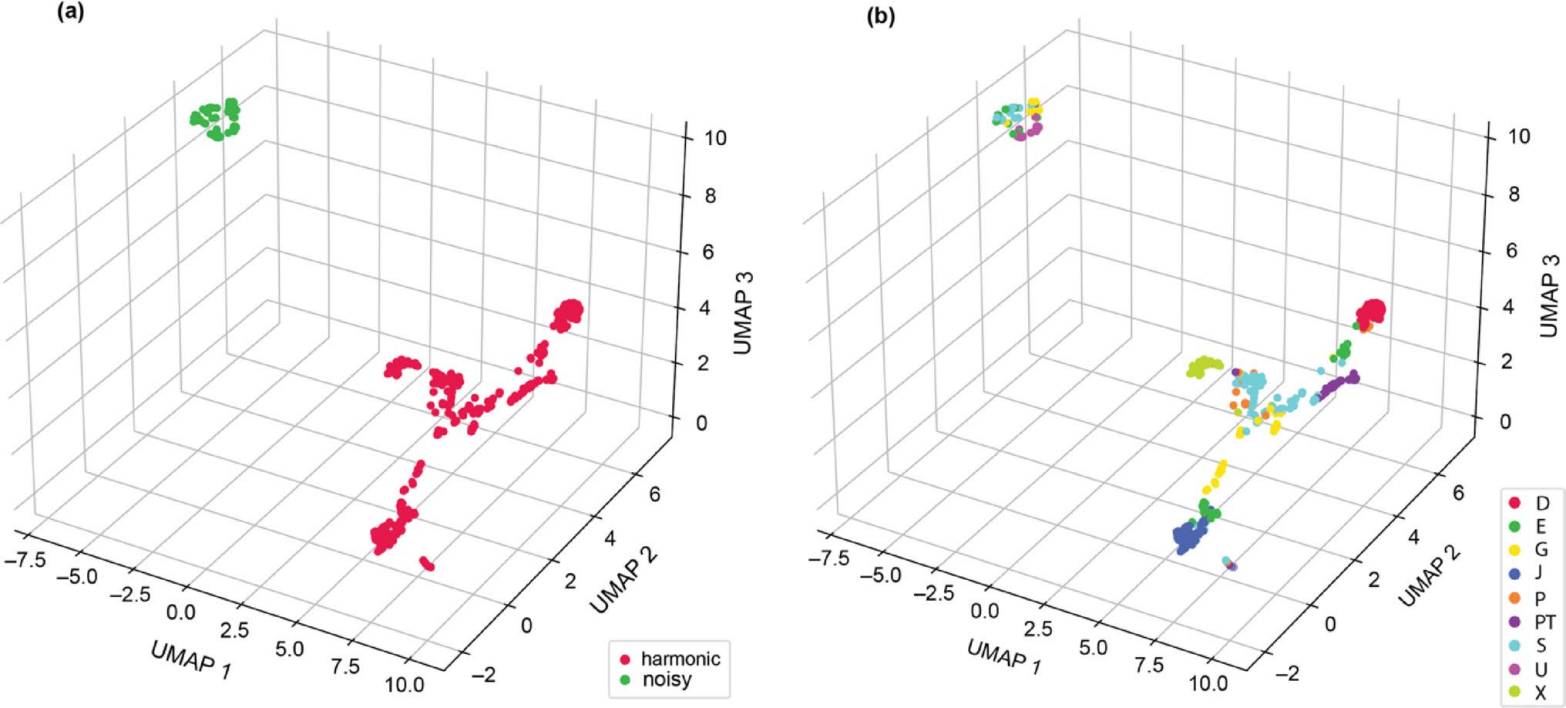
**(a)** Three-dimensional UMAP of two clusters selected base on unsupervised classification (elbow method) using spectrograms. Each dot in the latent space represents one tchak contact call, which were manually labelled to show two variants, namely the noisy (green) and the harmonic (red) variant. **(b)** Three-dimensional UMAP of manually labelled data points, showing each call of each individual in the dataset in latent space, colour-coded by individual identity

When manually adding individual labels to the data points of the two clusters (Fig. 2a), the second scatted cluster containing the harmonic tchak calls revealed strong individual separation (Fig. 2b). When using two clusters, nearest neighbour analysis (k_nn_=5) revealed a same-class probability of 100% (S_norm_=1.508) for having five nearest neighbours of the same class in the latent space. A Silhouette score of 0.768 indicated that calls within each variant of the contact call clustered closely together and separated well (see Fig. S6 in SI file).

We then used the acoustic features of the calls to assess whether calls could be classified to the correct subtype. Three important features were used (maximum f0, the time of minimum f0, and jitter; see Fig. S2 in SI file) to achieve 98.6% test accuracy (99.1% cross-validated), with f1-scores of 0.99 for the harmonic tchak calls, and 0.95 for the noisy tchak.

### Individual elements in harmonic and noisy Tchak contact calls

**Fig. 3:**
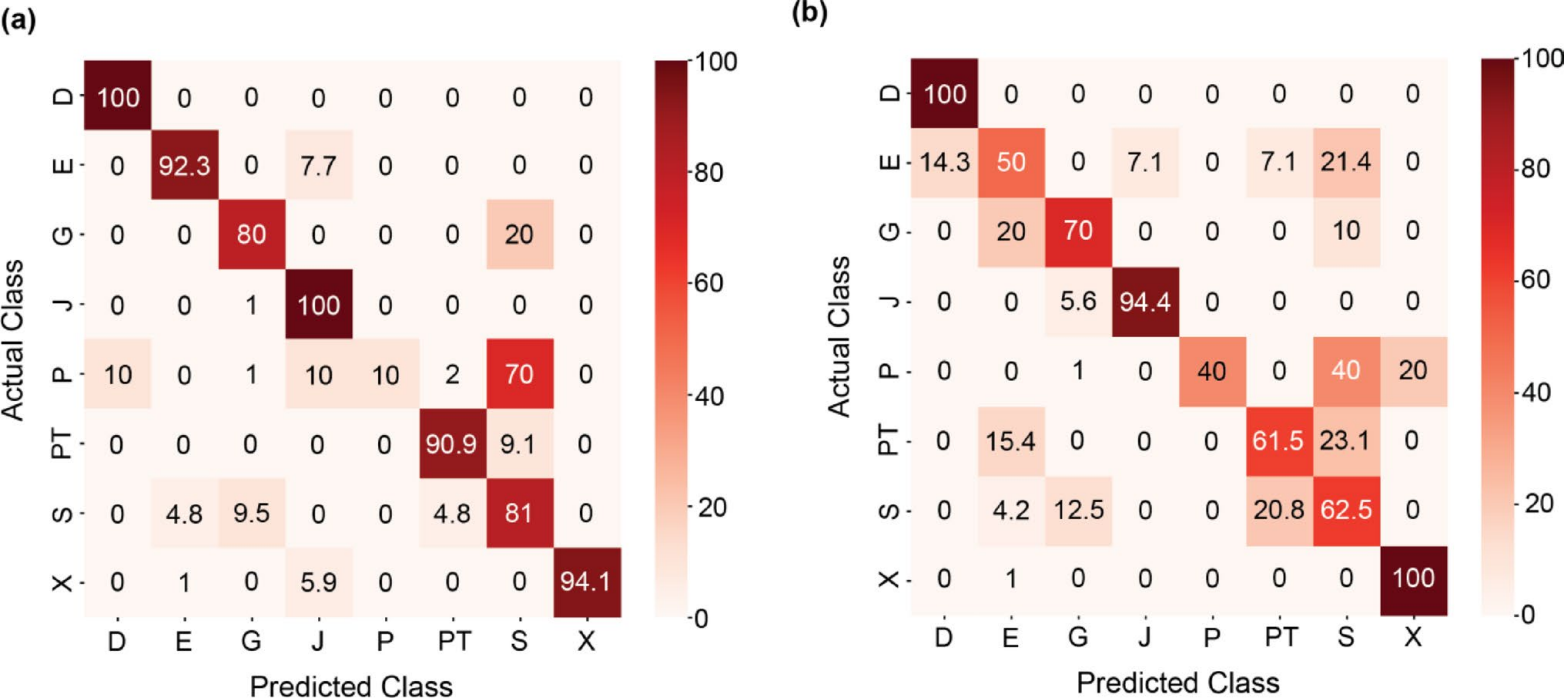
Confusion matrix showing actual and predicted class membership in percentage using (**a**) spectrograms and (**b**) acoustic features for individuals (capital letters) on the test dataset of harmonic tchak contact calls

Using 397 harmonic tchak calls of eight individuals, the CNN revealed 85.8% test accuracy when using the spectrograms for classification, with f1-score ranging between 0.33 and 0.97 (Fig. 3a; Table 1). Individual classification based on acoustic features was conducted using nine important features (see Fig. S3) and yielded a test accuracy of 76.7% (cross-validated: 77.6%, f1-score range: 054–0.97; Fig. 3b; Table 1), implying high individual distinctiveness in harmonic tchak contact calls, irrespective of the analysis method.

**Table 1:** Model performance and evaluation results for harmonic Tchak contact calls using spectrograms and acoustic features. The model tested predictions based on individual identity (ID) of the calling jackdaws

ID	Precision		Recall		f1-score		Support	
	Spectrogram	Acoustic	Spectrogram	Acoustic	Spectrogram	Acoustic	Spectrogram	Acoustic
D	0.96	0.92	1.00	1.00	0.98	0.96	25	25
E	0.92	0.58	0.92	0.50	0.92	0.54	13	13
G	0.67	0.64	0.80	0.70	0.73	0.67	5	5
J	0.86	0.94	1.00	0.94	0.92	0.94	18	18
P	1.00	1.00	0.10	0.40	0.18	0.57	10	10
PT	0.91	0.57	0.91	0.62	0.91	0.59	11	11
S	0.65	0.62	0.81	0.62	0.72	0.62	21	21
X	1.00	0.93	0.94	1.00	0.97	0.97	17	17

**Fig. 4:**
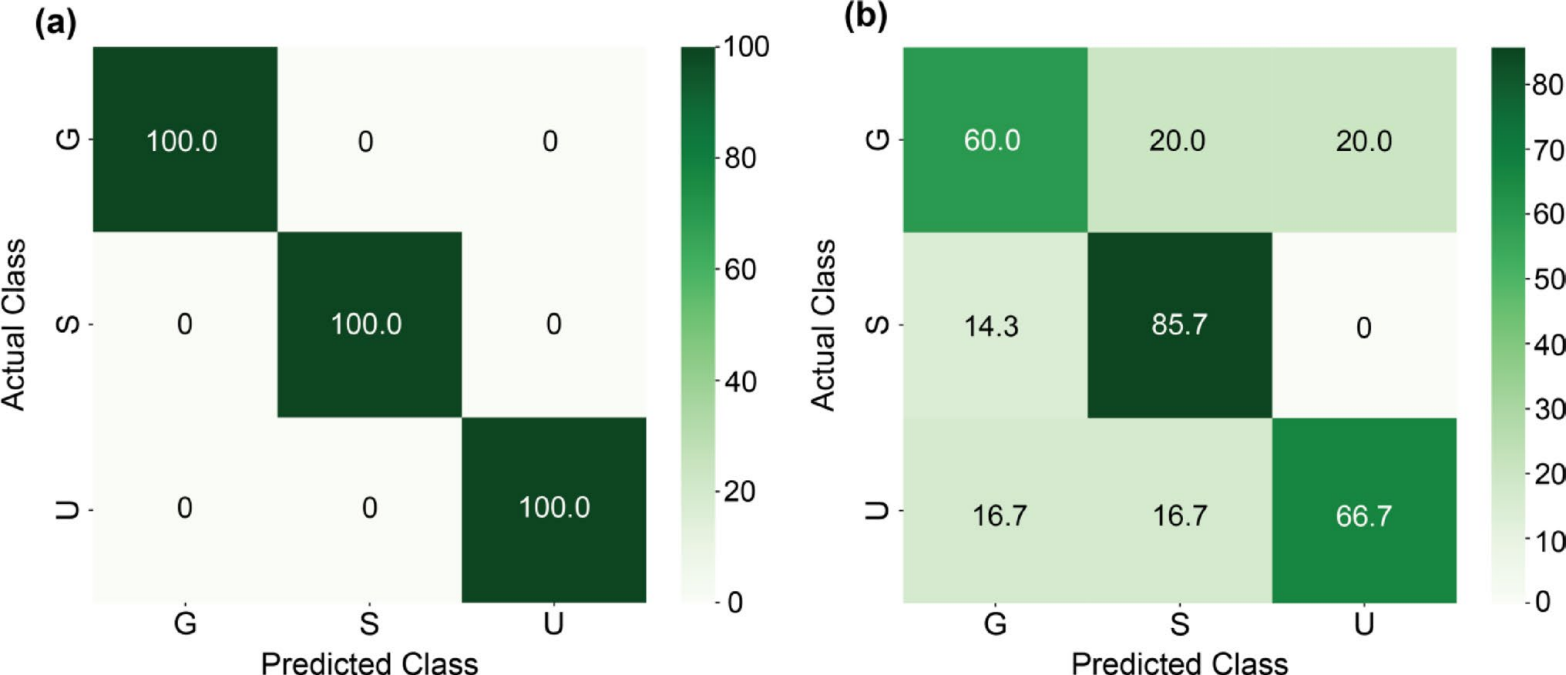
Classification results for individuals (indicated by capital letters) on the test dataset of noisy tchak contact calls. The confusion matrix displays actual class and predicted class membership in percentage for (**a**) spectrograms and (**b**) acoustic features

In the case of the noisy tchak calls (*N* = 57) recorded from three individuals, the test accuracy was 100% for spectrograms (Fig. 4a; Table 2). When testing for individuality based on acoustic features within noisy tchak calls, the test accuracy reached only 72.2% (cross-validated: 61.5%; Fig. 4b; Table 2), with f1-scores ranged between 0.60 and 0.80, using five important features (see Fig. S4 in SI file).

In sum, the classification using spectrograms yielded higher accuracies in identifying individual identities based on tchak contact calls than models based on acoustic features.

**Table 2:** Model evaluation results for noisy Tchak contact call analyses using acoustic features and spectrograms in comparison

ID	Precision		Recall		f1-score		Support	
	Spectrogram	Acoustic	Spectrogram	Acoustic	Spectrogram	Acoustic	Spectrogram	Acoustic
G	1.0	0.60	1.0	0.60	1.0	0.60	7	5
S	1.0	0.75	1.0	0.86	1.0	0.80	6	7
U	1.0	0.80	1.0	0.67	1.0	0.73	5	6

## Discussion

Our results show that the tchak contact calls of adult male jackdaws, while structurally variable, reliably encode individual identity in both its noisy and harmonic variants. Using both acoustic features and spectrogram-based analyses, we show that individual classification accuracy was consistently high, particularly when using spectrograms, which capture more of the call’s structural detail. These findings highlight the potential of structurally simple contact calls to convey socially relevant, individual-level information.

Both analytical approaches performed well, but spectrogram-based classification consistently yielded higher accuracies than models based on acoustic features. This suggests that spectrograms, as image-based representations of the full call structure, may better capture subtle yet informative aspects of individual vocal signatures, particularly in structurally complex or variable calls. In noisy tchak calls, which are characterized by low harmonicity and increased noise toward the end of the call, classification accuracy based on standard acoustic features was notably lower. Although we included features such as the proportion of voiced parts, jitter, and the harmonic-to-noise ratio, these features did not sufficiently capture the structural complexity required for accurate individual classification. This limitation underscores the potential value of richer signal representations. Similar findings were reported by Stowell et al. (2016), who used methods such as linear predictive coding (LPC) and adaptive discrete Fourier transforms (aDFT) to improve individual classification of jackdaw contact calls. Like our spectrogram-based approach, their methods retained more structural and temporal detail than standard acoustic measures, reinforcing the importance of these features for identifying individual vocal signatures.

The harmonic tchak variant exhibited greater acoustic variability than the noisy form, which was reflected in higher variation in both temporal and frequency parameters. Individual discrimination based on acoustic features was further based on duration, harmonicity, the time of maximum fundamental frequency, as well as fundamental frequency variability and pitch variability over time. Harmonic calls were also more widely scattered in the UMAP clustering in general (Fig. 3a and b), suggesting more structural variability. In contrast, noisy tchak calls exhibited a more consistent acoustic profile across individuals. This pattern may indicate that harmonic calls allow for more individual flexibility or context-sensitive modulation. Such flexibility aligns with recent work highlighting the cognitive and contextual flexibility of vocal production in corvids, which may support fine-tuned vocal responses to social environments (Liao et al. 2025). Notably, individuals that produced both variants (G, S, and E – with the latter excluded from the analysis of noisy tchak calls due to low sample size) showed greater acoustic dispersion in the harmonic tchak. Age is unlikely to explain this pattern, as both variants were recorded in birds hatched in 2005 and 2006 (see Fig. S7 in SI file).

We also considered whether social factors, such as pair bonding, might influence vocal similarity or variability. The ‘relationship intelligence’ hypothesis (Emery et al. 2007) predicts that lifelong monogamous pair bonds, like those observed in jackdaws, would favour vocal coordination and synchrony. Supporting this idea, Luef et al. (2017) found that monogamous raven pairs produced highly similar long-distance calls, potentially to enhance joint territorial defence. In contrast, we found no evidence of such a pattern in our data. Among the four paired individuals (P, S, U, X), call variant use was mixed: two produced only the harmonic variant, one produced only the noisy variant, and one produced both. Similarly, among the five unpaired individuals (D, E, G, J, PT), two produced only the harmonic variant, and three used both variants. Thus, our current data do not support the relationship intelligence hypothesis in this context. Broader sampling across additional flocks would be needed to assess whether these patterns are stable or artifacts of limited sampling.

Furthermore, we explored whether kinship influenced vocal similarity - that is, whether genetic or nest siblings would have more similar calls and thus be more frequently misclassified. Individuals J, X, and U were nest siblings (unrelated genetically, but raised together), while individuals D and P were genetic siblings. The remaining five individuals were unrelated and raised in separate nests. Misclassification patterns did not correspond with either genetic relatedness or shared developmental environment. Thus, these observations suggest that neither kinship nor social relatedness can explain the acoustic similarities or dissimilarities observed in our classification results.

Despite the differences in acoustic structure, both noisy and harmonic tchak calls could be individually discriminated based on shared acoustic features. Shared acoustic features included mean fundamental frequency, time on minimum fundamental frequency, the proportion of voiced parts, and jitter. These source-related features appear sufficient for individual-level discrimination. Although our study did not provide evidence for signature contact calls (i.e. calls used by individuals as consistent identity labels), these shared acoustic features provide great potential. Within the corvid family, individual vocal signatures have been shown in rooks, jungle crows, and American crows (Benti et al. 2019; Kondo et al. 2010; Mates et al. 2015). Signature contact calls are also widespread in other cognitively demanding fission-fusion societies (in parrots: Monk parakeets (*Myiopsitta monachus*; Smeele et al. 2023; Smith-Vidaurre et al. 2020), Green-rumped parrotlets (*Forpus passerines*; Berg et al. 2011); dolphins: Bottlenose dolphins (*Tursiops truncatus* and *T. aduncus*; Janik et al. 2006; Probert et al. 2023) and Spinner dolphins (*Stenella longirostris*; Rio 2023), highlighting the possible link between social and communicative complexity.

The presence of two structurally distinct variants within the tchak contact call raises intriguing questions about their functional significance. Since all calls were recorded while individuals were foraging on the ground, the tchak contact call, in general, may facilitate short-distance coordination or cohesion among group members or social partners during foraging. In other species, contact calls produced during foraging serve important roles in maintaining social cohesion, group coordination, and identity advertisement (reviewed in Kondo and Watanabe 2009). While we cannot yet determine the function of the harmonic versus noisy variants, their consistent deployment during foraging supports a social role, possibly in facilitating coordination within variable social settings. This may be particularly relevant in ecologically complex contexts such as loosely structured, mixed species flocks, known to occur frequently in corvids like jackdaws, crows, and rooks (Röell 1978; Andren 1992). In such groups, vocal signals may help maintain cohesion and coordination among individuals with varying degrees of social familiarity. Although flock composition was not directly assessed in this study, the possibility that tchak contact calls contribute to group coordination across different social or species boundaries merits future investigation. Further studies should also examine whether the two call variants are used flexibly by individuals depending on ecological or social circumstances.

Although our results support the distinction between noisy and harmonic tchak calls, we cannot entirely rule out potential seasonal or developmental influences. However, given that both call variants were recorded in the same season (compare Fig. S8 in SI file), using identical equipment, and were produced by individuals across both age groups, we interpret them as robust and biologically meaningful call type variants. Nonetheless, the limited sample size for the noisy variant constrains the strength of our conclusions, and further research should test whether call structure varies systematically with age, sex, dominance status, or ecological context. Behavioural experiments assessing whether receivers perceive or respond differently to harmonic versus noisy calls would help clarify their communicative function and relevance for individual recognition.

Taken together, our results indicate that the jackdaw tchak contact call contains consistent structural variation that may facilitate individual recognition during foraging and hence, may serve to maintain spatial proximity between social partners. More broadly, we show that even within a single call type, structural variation can coexist with robust identity cues. This combination may allow jackdaws to balance reliable individual recognition with context-sensitive communication, reflecting the demands of their socially and ecologically complex lives.

## Supplementary Information

Below is the link to the electronic supplementary material.

Supplementary Material 1

Supplementary Material 2

## Data Availability

Data was uploaded as a.zip file with the supplemental material and has been deposited in Zenodo under the DOI https://doi.org/10.5281/zenodo.17463735.
