# Supplementary material for: Variability and individuality in the contact calls of jackdaws (Corvus monedula)
Source: Anim Cogn. 2025 Dec 16;29(1):2. doi: 10.1007/s10071-025-02022-4 (PMC12705776; doi:10.1007/s10071-025-02022-4)
Supplement: Supplementary file 1 — Supplementary Material 1 [file 10071_2025_2022_MOESM1_ESM.docx]

**Supplementary Information (SI) as referred to in**

# **Variability and Individuality in the Contact Call of Jackdaws (*Corvus monedula*)**

Georgine Szipl, Anton Baotic & Kurt Kotrschal

**Method Section**


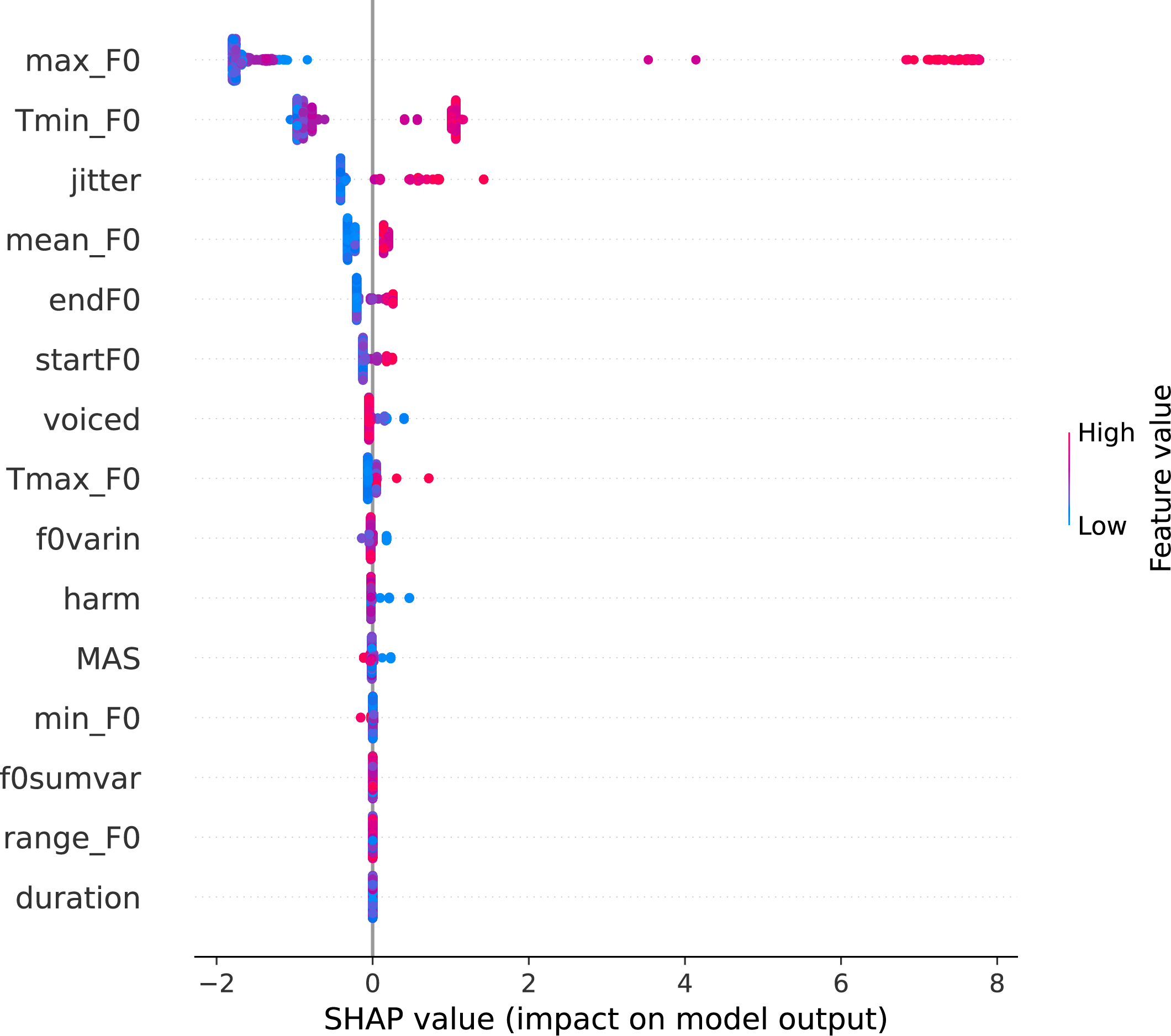


**Fig. S1**: Selected acoustic parameters for the two variants of tchak contact calls based on SHAP value > 1. The three acoustic features that were selected were maximum fundamental frequency, the time of minimum fundamental frequency, and jitter. For description of the abbreviations of acoustic features, see Table S1


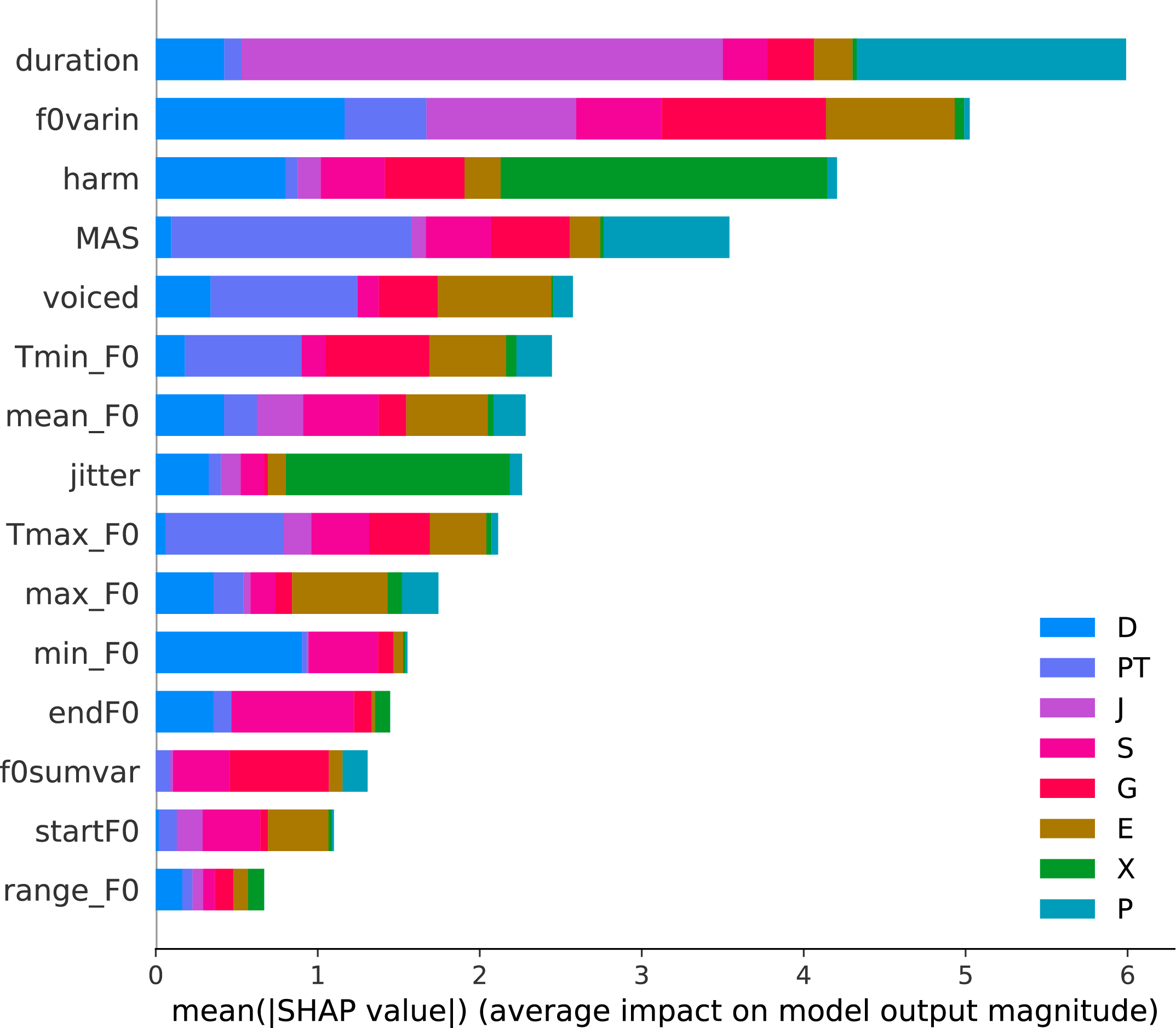


**Fig. S2**: Selected acoustic features used in the model to discriminate individuals based on the acoustic features of harmonic tchak contact calls. Colours indicate individuals, capital letters denote individual identity. Selection of features was based on SHAP values > 2. Abbreviations of acoustic features can be found in Table S1


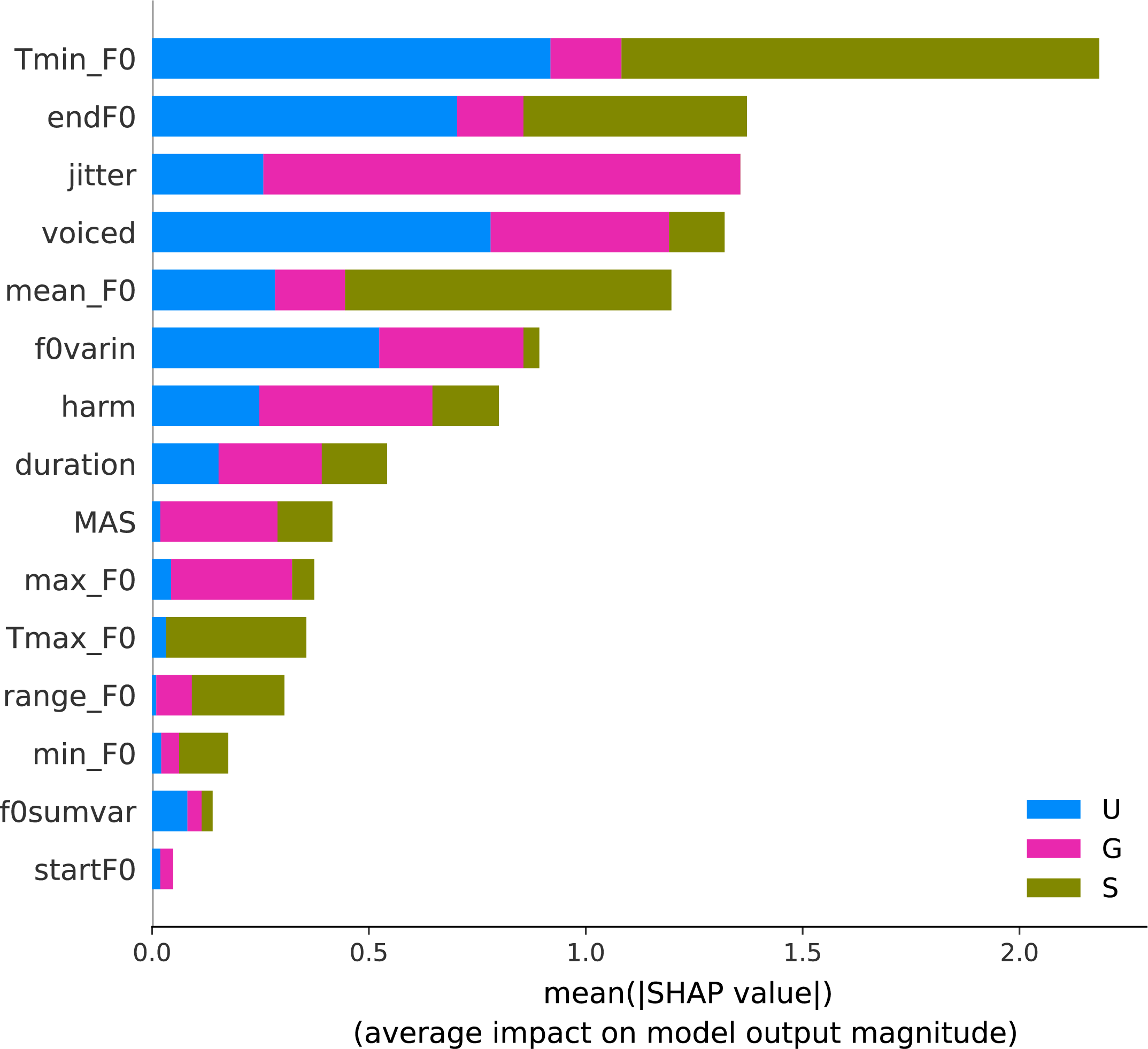


**Fig. S3**: Selected acoustic features used in the model to discriminate individuals (color-coded with capital letters indicating individual identity) based on the acoustic features of noisy tchak contact calls. Selection is based on SHAP values > 1. Abbreviations of acoustic features are described in Table S1

**
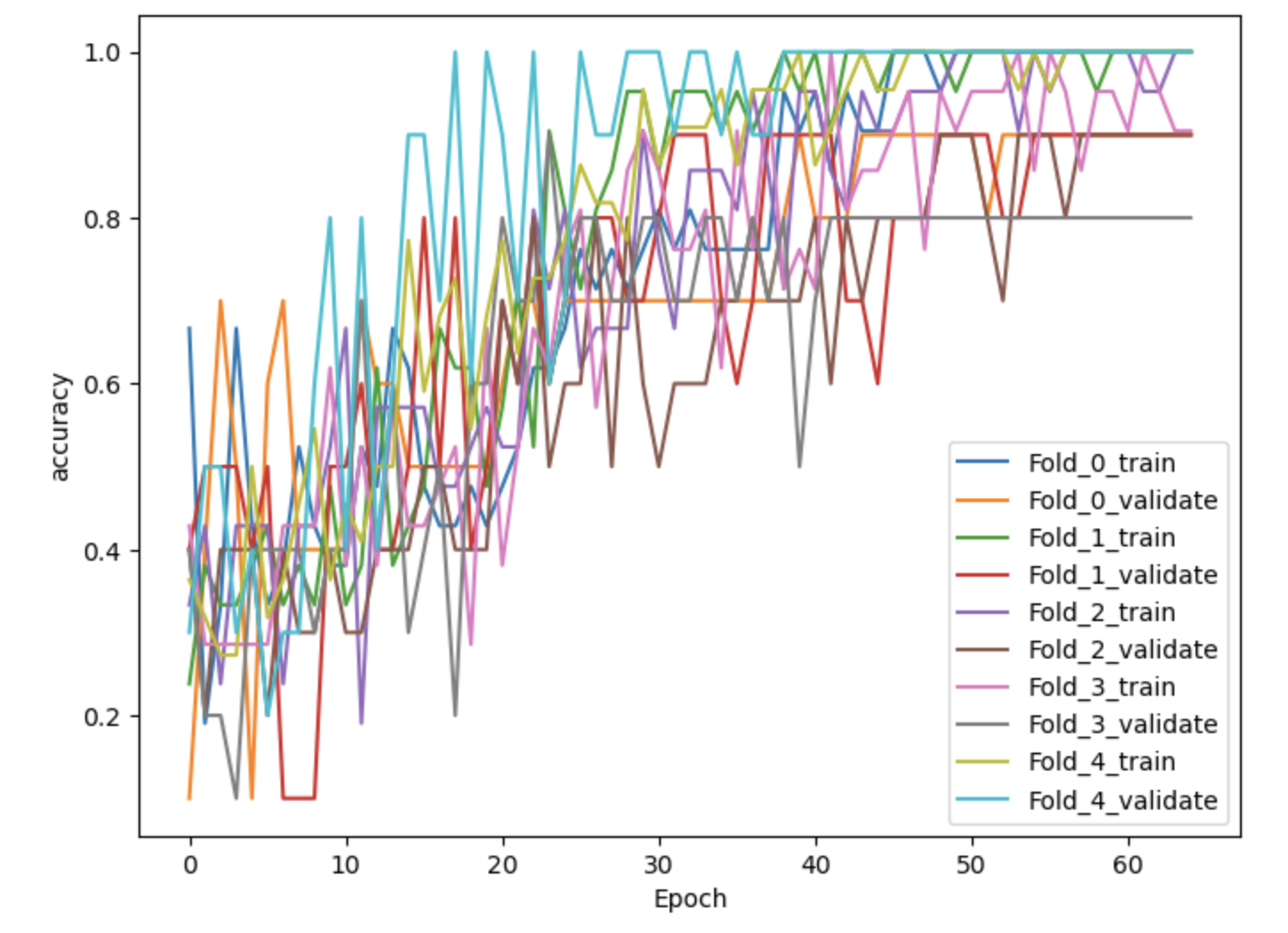
**

**Fig. S4**: Learning curve for noisy tchak contact calls using supervised convolutional neural network (CNN) to classify individuals based on spectrograms. The validation and training in five folds achieved a test accuracy of 100% on the test dataset


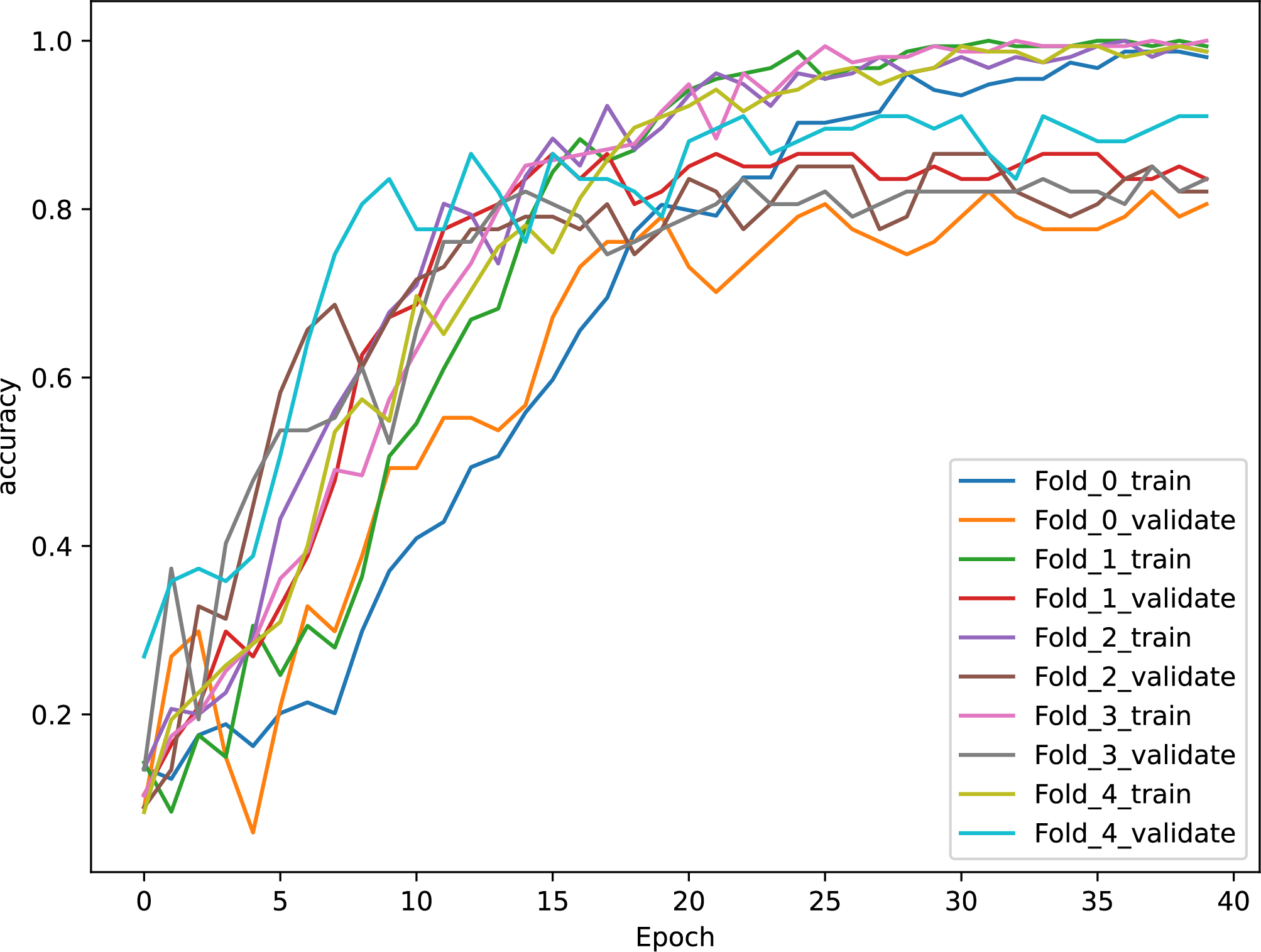


**Fig. S5**: Learning curve for harmonic tchak contact calls using supervised convolutional neural network (CNN) to classify individuals based on spectrograms. The validation and training in five folds achieved a test accuracy of 85.8% on the test dataset


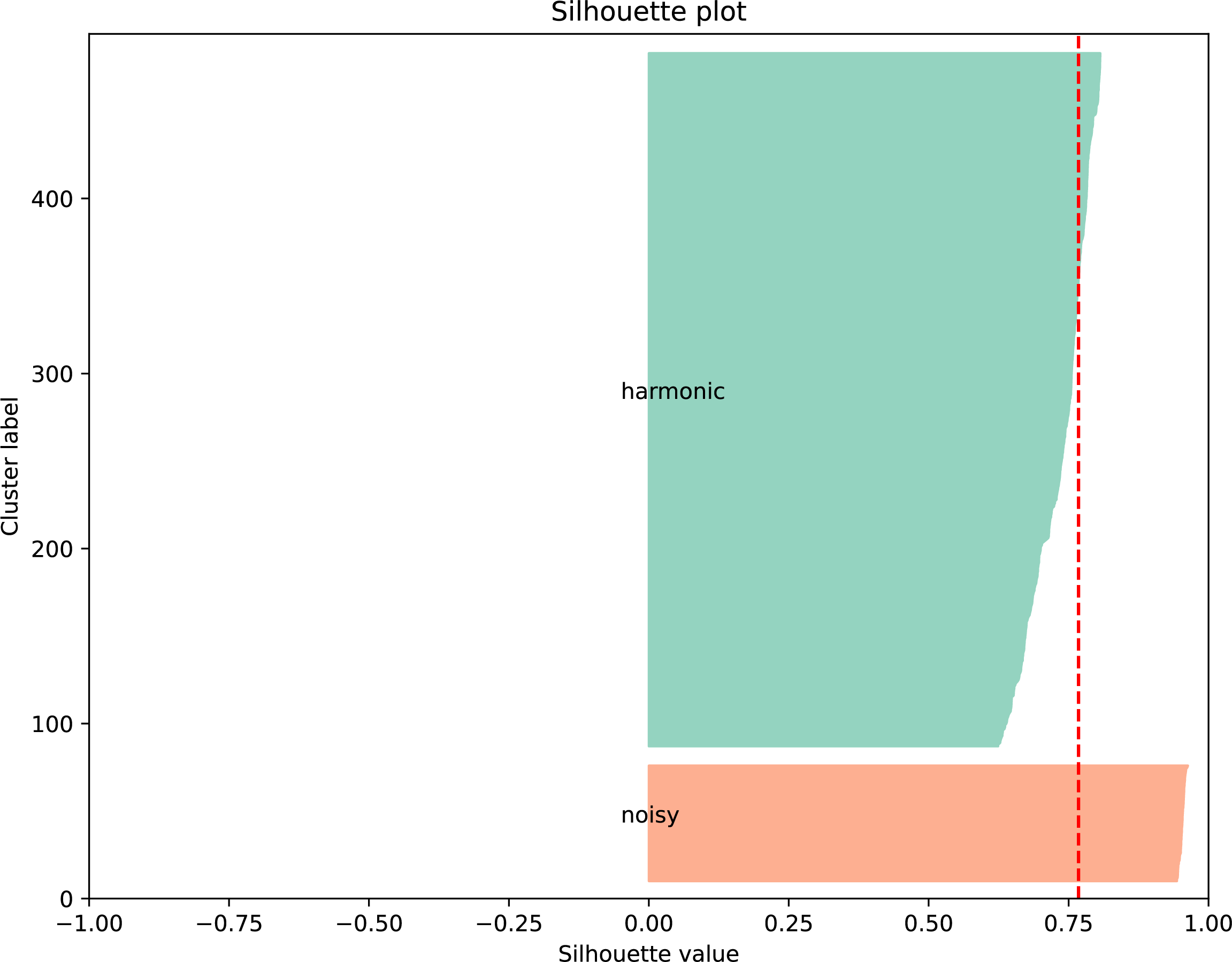


**Fig. S6**: Silhouette plot showing the degree of separation of the two clusters (call variants of the tchak contact call). The silhouette value of 0.768 indicated that the noisy variant is far away from the neighbouring harmonic cluster


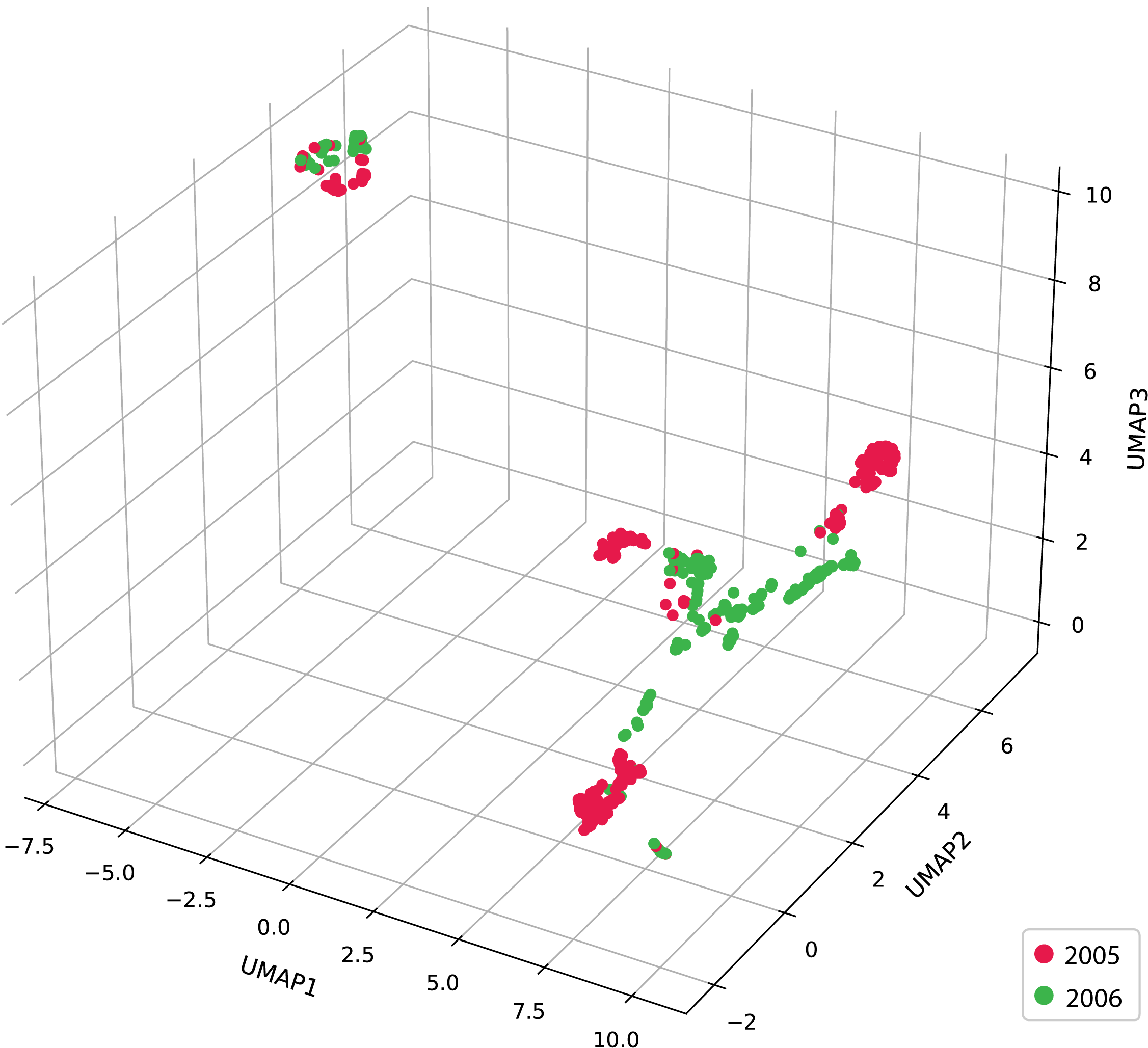


**Fig. S7**: Three-dimensional UMAP of manually labelled data points, showing each call of each individual in the dataset in latent space, colour-coded by the hatching year of the individuals


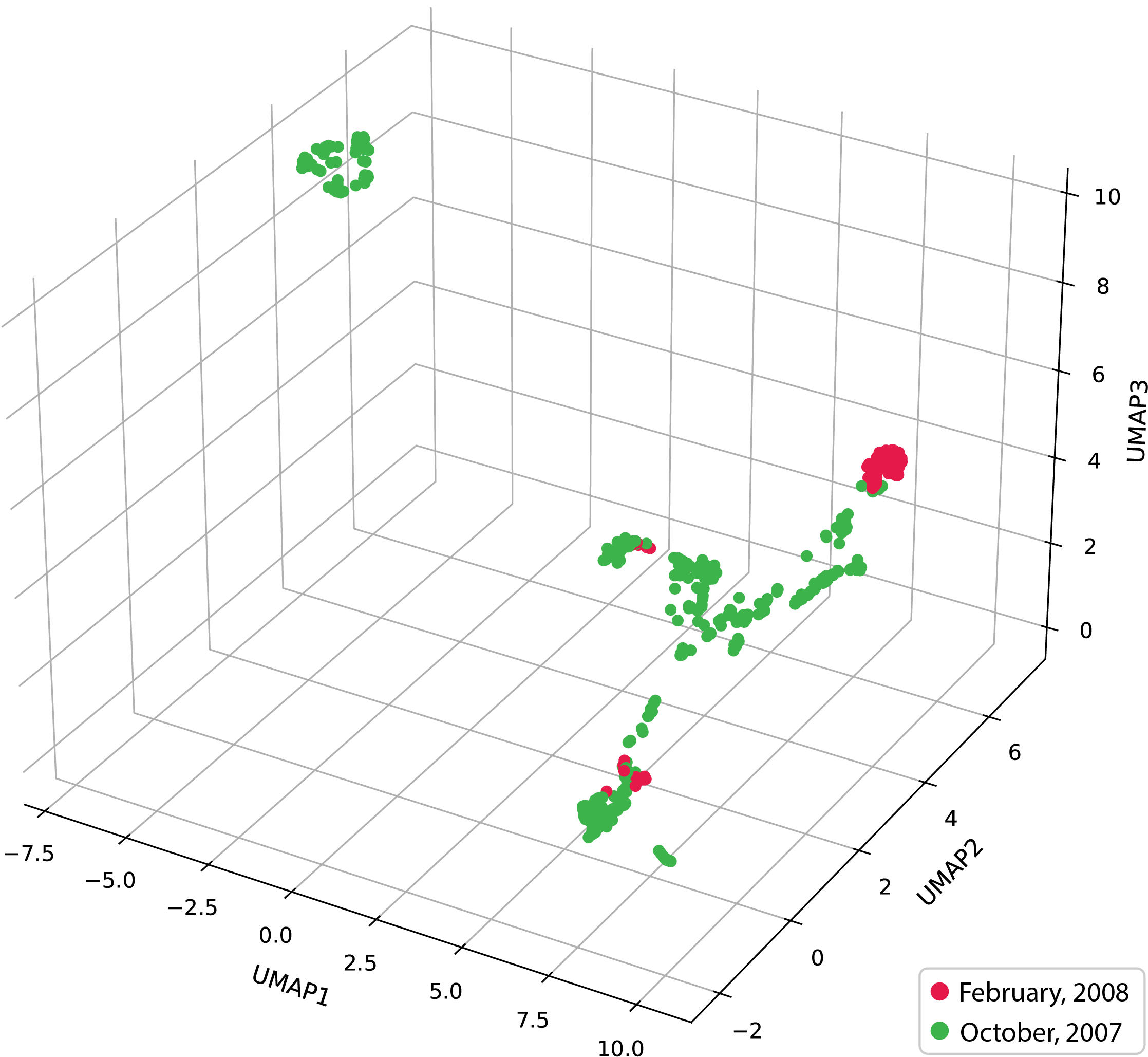


**Fig. S8**: Three-dimensional UMAP of manually labelled data points, showing each call of each individual in the dataset in latent space, colour-coded by recording season

**Supplemental Table S1**: Acoustic features measured in this study with definitions of abbreviations.

| **Measured acoustic parameters** | **Definition** |
| --- | --- |
| **Temporal parameters** | |
| duration | Total duration of the sound file in seconds. |
| Tmax_F0 | Time (in seconds) at which the maximum F0 occurs within the sound file. This indicates the temporal position of the highest pitch. |
| Tmin_F0 | Time (in seconds) at which the minimum F0 occurs within the sound file. This indicates the temporal position of the lowest pitch. |
| **Fundamental frequency related parameters** | |
| mean_F0 | Mean fundamental frequency over the voiced portion of the sound, measured in Hertz (Hz). Fundamental frequency refers to the lowest frequency of a periodic waveform, representing the pitch of the sound. |
| max_F0 | Maximum fundamental frequency detected within the voiced segments, in Hz. |
| min_F0 | Minimum fundamental frequency detected within the voiced segments, in Hz. |
| range_F0 | Difference between the maximum and minimum fundamental frequency values, representing the pitch range of the vocalization, in Hz. |
| voiced | Proportion of the total duration that is classified as voiced, indicating the proportion of time the vocal folds are vibrating. |
| startF0 | Fundamental frequency at the onset of the vocalization, in Hz. |
| endF0 | Fundamental frequency at the end of the vocalization, in Hz. |
| f0sumvar | Average fundamental frequency variation per unit time |
| f0varin | Index of fundamental frequency variability, often calculated as the standard deviation of the fundamental frequency normalized by its mean, providing a measure of relative pitch variability. |
| MAS | Mean absolute slope; measures pitch variability over time |
| jitter | Measure of frequency variation from cycle to cycle in the signal, representing the stability of the pitch. Higher jitter values indicate greater instability, often perceived as roughness in the sound. |
| **Spectral parameter** | |
| harm | Harmonics-to-Noise Ratio (HNR); measures the ratio of periodic (harmonic) components to noise components in the signal, indicating voice quality. Higher values suggest clearer, more tonal sounds, while lower values indicate noisier signals. |

**Supplemental Table S2**: Descriptive statistics of the most prominent acoustic features of tchak contact calls in jackdaws, separately for the two variants found (harmonic and noisy) and for each individual (ID) within each variant, and in total. Numbers indicate mean±SD, bold numbers are mean±SD for each variant and all calls in total (n=463), total sum is the mean±SD for the total number of calls. Numbers in brackets indicate individual sample sizes. One noisy thack call of one individual (J) was removed.

| **Subtype** | **ID** | **duration** | **mean f0** | **harm** | **voiced** | **f0sumvar** | **jitter** | **max_F0** | **Tmin_F0** |
| --- | --- | --- | --- | --- | --- | --- | --- | --- | --- |
| **Harmonic (n=397)** |  | **0.155 ± 0.023** | **685.42 ± 103.07** | **9.29 ± 3.16** | **0.83 ± 0.16** | **4757.91 ± 955.95** | **0.027 ± 0.012** | **1133.91 ± 89.21** | **0.129 ± 0.031** |
|  | D (73) | 0.142 ± 0.007 | 701.96 ± 86.83 | 8.08 ± 1.31 | 0.78 ± 0.12 | 4496.51 ± 1052.79 | 0.025 ± 0.005 | 1076.89 ± 107.22 | 0.113 ± 0.020 |
|  | E (45) | 0.154 ± 0.014 | 625.31 ± 55.69 | 10.31 ± 1.33 | 0.93 ± 0.05 | 4845.43 ± 693.23 | 0.023 ± 0.005 | 1098.49 ± 95.02 | 0.131 ± 0.020 |
|  | G (34) | 0.147 ± 0.013 | 695.71 ± 131.18 | 9.85 ± 1.76 | 0.82 ± 0.16 | 5169.65 ± 1178.01 | 0.026 ± 0.007 | 1160.26 ± 51.84 | 0.117 ± 0.027 |
|  | J (61) | 0.190 ± 0.012 | 681.65 ± 123.58 | 12.06 ± 2.03 | 0.90 ± 0.13 | 4431.48 ± 814.77 | 0.018 ± 0.004 | 1182.32 ± 36.18 | 0.161 ± 0.032 |
|  | P (16) | 0.126 ± 0.008 | 787.62 ± 105.81 | 9.30 ± 3.55 | 0.62 ± 0.23 | 5310.48 ± 1205.57 | 0.027 ± 0.005 | 1165.28 ± 41.45 | 0.089 ± 0.020 |
|  | PT (42) | 0.160 ± 0.022 | 662.22 ± 86.02 | 10.68 ± 2.40 | 0.95 ± 0.05 | 4606.42 ± 473.32 | 0.023 ± 0.010 | 1129.83 ± 74.88 | 0.148 ± 0.022 |
|  | S (80) | 0.149 ± 0.025 | 661.64 ± 103.36 | 10.00 ± 2.94 | 0.89 ± 0.12 | 5079.11 ± 1069.73 | 0.024 ± 0.008 | 1118.56 ± 93.55 | 0.135 ± 0.023 |
|  | X (46) | 0.152 ± 0.012 | 742.41 ± 59.02 | 3.62 ± 1.29 | 0.63 ± 0.17 | 4603.15 ± 685.09 | 0.053 ± 0.012 | 1194.91 ± 56.83 | 0.102 ± 0.017 |
| **Noisy (n=66)** |  | **0.165 ± 0.011** | **850.55 ± 85.96** | **5.62 ± 2.00** | **0.64 ± 0.27** | **5797.72 ± 884.25** | **0.055 ± 0.014** | **1424.60 ± 81.98** | **0.150 ± 0.020** |
|  | E (9) | 0.163 ± 0.012 | 819.06 ± 33.70 | 3.91 ± 1.66 | 0.65 ± 0.21 | 6232.28 ± 401.22 | 0.062 ± 0.013 | 1461.38 ± 44.30 | 0.153 ± 0.009 |
|  | G (16) | 0.163 ± 0.007 | 846.17 ± 34.24 | 5.98 ± 0.92 | 0.73 ± 0.26 | 5852.26 ± 345.57 | 0.059 ± 0.005 | 1415.62 ± 24.11 | 0.153 ± 0.007 |
|  | S (22) | 0.173 ± 0.007 | 819.54 ± 31.33 | 5.74 ± 2.41 | 0.71 ± 0.13 | 5903.41 ± 530.95 | 0.052 ± 0.011 | 1439.02 ± 62.55 | 0.161 ± 0.006 |
|  | U (19) | 0.158 ± 0.011 | 905.06 ± 138.67 | 6.00 ± 2.01 | 0.48 ± 0.35 | 5423.57 ± 1437.03 | 0.052 ± 0.019 | 1398.05 ± 128.85 | 0.134 ± 0.030 |
| **Total sum** |  | **0.157 ± 0.022** | **708.96 ± 116.13** | **8.77 ± 3.29** | **0.81 ± 0.19** | **4906.13 ± 1012.79** | **0.031 ± 0.016** | **1175.35 ± 134.60** | **0.132 ± 0.030** |
